# Concealed Fertility and Extended Female Sexuality in a Non-Human Primate (*Macaca assamensis*)

**DOI:** 10.1371/journal.pone.0023105

**Published:** 2011-08-10

**Authors:** Ines Fürtbauer, Michael Heistermann, Oliver Schülke, Julia Ostner

**Affiliations:** 1 Primate Social Evolution Group, Courant Research Centre Evolution of Social Behaviour, Georg-August-University Göttingen, Göttingen, Germany; 2 Reproductive Biology Unit, German Primate Center, Göttingen, Germany; 3 Integrative Primate Socioecology Group, Max Planck Institute for Evolutionary Anthropology, Leipzig, Germany; 4 Courant Research Centre Evolution of Social Behaviour, Georg-August-University Göttingen, Göttingen, Germany; Indiana University, United States of America

## Abstract

In numerous primates living in mixed-sex groups, females display probabilistic cues of fertility to simultaneously concentrate paternity to dominant males while diluting it amongst others as a means to reduce the risk of infanticide and to increase male care for offspring. A few species, however, lack these cues and potentially conceal fertility from males; yet, to date, little is known about mating patterns and their underlying proximate mechanisms in such species. Here, we investigated mating activity and sexual consortships relative to female reproductive state in wild Assamese macaques (*Macaca assamensis*), a species where females lack prominent anogenital swellings and copulation calls. During two mating seasons (2837 contact hours) we recorded sexual and social behaviors, sexual consortships, and collected 1178 fecal samples (n = 15 females) which were analyzed for progestogen concentrations to assess female reproductive state and to determine the timing of ovulation and conception. Although mostly conceiving in their first ovarian cycle, females were sexually receptive throughout the entire 4-month mating season, and within-cycle mating frequencies were not increased during fertile phases. Dominant males did not monopolize fertile matings, and consortships by high-ranking males lasted for long periods, which were not exclusively linked to female fertile phases. Furthermore, females copulated promiscuously but not randomly, i.e. for almost every female, matings were concentrated to a certain male, irrespective of male rank. Collectively, we demonstrate that fertility is undisclosed to males. The extreme extended female sexuality facilitated by concealed fertility may allow females to create differentiated mating relationships within a promiscuous mating system. Our study provides important new insight into the plasticity of female sexuality in non-human primates.

## Introduction

Sexual selection theory states that due to the asymmetry in parental investment, males should increase their number of offspring by mating with many females whereas females should mate more selectively in order to increase their reproductive success [1], [2]. Contrary to these predictions, however, female promiscuity is widespread among vertebrates and invertebrates [3]–[7]. Given the potential costs of this behavior in terms of time, energy, disease and parasite transmission, predation and/or injury inflicted by males [3], [8]–[13], females are expected to gain compensatory benefits from mating with many males. Polyandrous mating has therefore commonly been associated with procreative benefits as it may ensure insemination, promote sperm competition, increase the genetic variability of offspring, or avoid inbreeding [6], [8], [14]–[17].

Polyandrous mating during non-fertile stages, however, must serve functions other than reproduction. The most convincing non-procreative explanation (among several hypotheses; see [7]) is that female polyandrous mating is a female counterstrategy to infanticide risks posed by males [6], [7], [18]–[20]. Primates, in particular, face a high risk of infanticide owing to their slow life histories [6]. It has been proposed that by mating with many males females may manipulate male paternity assessment as a means to reduce infanticide risks [18], [21]–[27]. Male primates usually base their decision on whether or not to attack an infant on paternity estimates, and thus should be less likely to attack infants of females they have previously mated with [28], [29]. Additionally, if paternity estimates are high for certain males, polyandrous mating may secure or increase male care from these males for future infants including protection from infanticide [25], [28], [30]–[32].

At a proximate level, polyandrous mating requires that females cannot be monopolized by a single (dominant) male. Female primates exhibit a number of physiological, morphological and behavioural alterations in order to break the dominant male's monopoly, i.e. to decrease male mating skew (reviewed in [33]). Old World primates, in particular, exhibit prolonged mating periods within the ovarian cycle, which has been attributed to their long follicular phases [25]. The timing of ovulation varies within these periods, and thus, is unpredictable for males, hence enabling females to mate with multiple partners [25]. At the same time, females of numerous Old World primates exhibit anogenital (sexual) swellings and/or copulation calls, i.e. cues that enhance female attractivity around the probable timing of ovulation, and thus, in some way, advertise rather than obscure it [34], [35].

Numerous studies focussing on these traits have shown that they function as “graded signals” of ovulation probability, enabling females to overcome the “female dilemma” [36], i.e. on the one hand, to concentrate paternity in dominant males, while on the other hand, confusing it among many males [35]–[41]. In some species, however, females lack these cues [34], raising the question whether these females, nevertheless, concentrate paternity in certain males. Unfortunately, to date, for many of these species little is known about female sexuality relative to reproductive state and the underlying proximate mechanisms involved. This study aims to address this important gap in our general understanding of mating systems and reproductive strategies in primates.

Assuming that paternity manipulation may be reached on different levels, the following two scenarios are possible in the absence of overt cues to fertility: (1) Females may exhibit olfactory (e.g. [42]) or behavioral indicators of reproductive state (i.e. “behavioral estrus”; [34], [43]) leading to a similar outcome, i.e. concentration (dominant males) when fertilization is most, and dilution (subordinate males) when it is less likely, respectively (see above). (2) Fecundity may be undisclosed to males, hence preventing monopolization by dominant individuals during fertile periods. Assuming that males, in the latter scenario, are unable to assess when ovulation is likely to be imminent, they must rely on mating history as their only estimate for paternity probability (instead of relating it to female attractivity and ovulation probability). As such, females may be freer but could still show (non-dominant based) mating biases towards certain males, i.e. matings may be distributed non-randomly across mating partners (see [28]).

Assamese macaques (*Macaca assamensis*) provide an excellent model to investigate the proximate mechanisms underlying female reproductive strategies in the context described above. This species belongs to the minority of macaque species [44] where females lack pronounced sexual swellings [45], [46] and copulation calls (Fürtbauer, pers. obs.). Assamese macaque females mostly conceive during their first ovarian cycle within the 4-month mating season [46]. Despite a clear male dominance hierarchy, male reproductive skew is low [47]–[49], indicating that males are unable to monopolize fertile females. Males in this species, however, frequently interact with infants (Ostner & Schülke, unpubl. data; Fürtbauer, pers. obs.) suggesting, according to recent modeling, high paternity estimates [28]. Yet, there is no information on the characteristics and patterns of female sexual activity in this species, which would provide new insight into female sexuality in primates in general.

Here, we investigate female mating activity and male consortships (as a marker of the males' ability to monopolize fertile females) in a wild group of Assamese macaques and relate it to female reproductive state, as determined from non-invasive fecal hormone analysis, to address (i) how female receptivity is distributed relative to reproductive state, (ii) whether females exhibit behavioral estrus (indicated by increased mating frequencies and male monopolization around ovulation), or whether fertility is undisclosed to males, and (iii) whether female matings are non-randomly distributed across males.

## Methods

This study was carried out in the field with wild monkeys and was completely non-invasive. Approval and permission to conduct research was granted by the authorities of Thailand (permit no. 0004.3/3618), and all research was undertaken in strict accordance with the ABS/ASAB guidelines for the ethical treatment of animals in research, the recommendations of the Weatherall report on the use of non-human primates in research, and the laws set forth by the National Research Council of Thailand and the regulations of the Department of National Parks, Wildlife and Plant Conservation, Bangkok, as well as the guidelines of the involved institutes.

### Study site and animals

The study was carried out at the Phu Khieo Wildlife Sanctuary (157,300 ha, 16°5′–35′N, 101°20′–55′E, 300–1,300 m a.s.l.), north-eastern Thailand, which consists of dry evergreen forest with patches of dry dipterocarp forest and bamboo stands [50]. During two consecutive mating seasons (MS = months during which conceptions may occur) data were collected on a fully habituated group (AS) of Assamese macaques. During the MS 2007/2008 (Oct 1^st^–Jan 31^st^) the study group comprised 53 individuals including 12 adult females and 13 males (8 adult and 5 large subadult males; for age classes see [47]). During the MS 2008/2009 (Oct 1^st^–Feb 13^th^) the group consisted of 55 individuals including 14 adult females and 15 males (10 adult and 5 large subadult males). We selected those females which, based on their reproductive history, were expected to conceive in the respective mating season. Thus, data are presented for 15 females which conceived during the respective mating season (11 multiparous and 4 primiparous females; Table 1). In the MS 2007/2008 none of the non-focal females conceived whereas in the MS 2008/2009 two females (fs08 and fs11) conceived unexpectedly and thus, had not been sampled.

**Table 1:** Study females, dominance rank, parity, and number of fecal samples for hormone analysis.

Mating season	Female	Dominance Rank^a^	Parity	Number of samples
2007/2008	fs03	3	mp	72
	fs05	2	pp	73
	fs06	4	mp	66
	fs07	10	mp	70
	fs08	1	mp	76
	fs10	6	mp	75
	fs11	9	mp	79
2008/2009	fs02	13	mp	76
	fs04	14	mp	72
	fs09	9	mp	79
	fs13	6	mp	85
	fs14	4	pp	88
	fs15	10	pp	80
	fs18	2	pp	93
	fs19	8	mp	94
**Total**				**1178**

a calculated separately for each mating season.

mp = multiparous; pp = primiparous.

### Behavioral observations

The study group was followed daily from dawn until dusk during the two mating seasons resulting in 2,837 contact hours with the animals (mean±sd: 11.1±0.7 contact hours per day). Various researchers and assistants (2.5±0.7 observers per hour; all trained by IF) collected data on social and sexual interactions using ad libitum and focal animal sampling (20–30 min; evenly distributed throughout the day and across females), which were pooled for further analyses and to determine for each female whether she was sexually receptive (i.e. mating regardless of fertility) on a given day. For all observed copulations (n = 1280) we noted the date, the time, the identities of the individuals, and whether the male ejaculated or not. Ejaculatory copulations could easily be distinguished from non-ejaculatory copulations due to a typical and pronounced pre-ejaculatory pause in male pelvic thrusts. In addition, if observed, we noted whether copulations were male- or female-initiated using approach behavior (<1 m) immediately prior to the mating as criterion. Also, we recorded whether females presented their hindquarters to the male in order to initiate copulations. In order to control for potential rank effects on mating behaviour (e.g. [34]), female dominance rank was established based on the exchange of clear submissive signals, i.e. “silent bared teeth” [51] and “make room” [47].

To indirectly assess the males' ability to assess female reproductive state, and hence to monopolize fertile females, we collected data on sexual consortships, i.e. spatio-temporal associations between a male and a female. Consorts were characterized by the pair staying in close proximity (<10 m), coordinating their movements, grooming, and mating. Consorts lasted from 1 to 49 days.

### Hormone analysis

Fecal hormone analysis to assess female reproductive state and timing of ovulation and conception has been described in detail by Fürtbauer et al. [46]. In brief, we collected on average 4.6±0.5 samples per week directly after defecation from each selected female (1178 samples in total, Table 1). Samples were stored at −20°C until hormone analysis. Following hormone extraction from freeze-dried and pulverized samples [46], fecal extracts were measured for concentrations of progestogens (20α-dihydroprogesterone; 20α-OHP) using a validated microtiterplate enzyme immunoassay (EIA; [46]). Sensitivity of the assay at 90% binding was 1.5 pg. Intra- and interassay coefficients of variation, calculated from replicate determinations of high- and low-value quality controls (made from 20α-OHP standard) were 7.5% and 13.1% (high) and 9.2% and 16.7% (low).

### Assessment of fertile phases and conceptions

As described previously [46], we determined the timing of ovulation/conception in our study females by using the defined post-ovulatory rise in fecal progestogen levels, taking into account the fecal excretion lag time. This approach has been successfully applied for monitoring female reproductive status, including timing of ovulation/conception and the female fertile phase, in a variety of primate species in a wide range of taxa [52]–[59]. We considered day −3 relative to the day of the defined progestogen increase (day 0) as the most likely day of ovulation/conception. Because of the possible variability in the temporal relationship between ovulation and the post-ovulatory fecal progestogen rise [60], and since daily samples during the peri-ovulatory period were not always available, data on the timing of ovulation/conception can be expected to include an error of 1–2 days. We defined the fertile phase as a 5-day period including days −2 and −3 (relative to day 0) plus the three preceding days to account for sperm longevity in the female reproductive tract [61].

### Data analyses

Non-parametric tests were applied using SPSS 17.0 (SPSS Inc.), with the level of significance set at alpha = 0.05. All statistical tests were two-tailed. Female dominance hierarchy was assessed using the I&SI method as implemented in MATMAN™ 1.1.4 (Noldus 2003) based on 375 agonistic interactions. Due to the death of one female (fs03) in June 2008, and the addition of three primiparous females to the data set in 2008/2009 (fs14, fs15, and fs18), both mating seasons were treated separately (Table 1).

To determine differences in daily copulation frequencies relative to reproductive state we performed two Generalized Linear Mixed Model (GLMM) analyses with Poisson-distributed error variances in R ([62]; package lme4). As assumed by the models, overdispersion was not significant. We used reproductive state and female dominance rank as predictor variables. In the first model, encompassing the entire mating season, reproductive states were defined as follows: “n” (non-fertile = acyclic and cyclic stages prior to conception; i.e. the period between the first observed copulation and the day before the onset of the fertile phase leading to conception), “fert” (fertile = the 5-day fertile phase), and “p” (pregnant = the period between the first day after the fertile phase of the conception cycle and the last observed copulation). In order to investigate within-cycle differences in daily copulation frequencies we performed a second model for which reproductive states were defined as follows: “pre” (prefertile = the five days preceding the fertile phase), “fert” (fertile = the 5-day fertile phase), and “post” (postfertile = the five days following the fertile phase). In both models, we included “female ID” and “mating season”, i.e. either 2007/2008 or 2008/2009 as random factors, and applied dummy-coding using “fert” as reference category.

To investigate the distribution of matings across males we used Nonacs's skew calculator (2003). We computed B indices and probability levels to test whether observed B values are due to random chance [63]. B values of 0 indicate randomly distributed mating, negative values suggest mating is more equally shared than random and positive values indicate greater than random skew.

## Results

### Female ovarian cycles and conceptions

Out of 15 study females, twelve conceived during the first and three during their second ovarian cycle (all three in 07/08). For 13 females we could hormonally determine the precise timing of their fertile phases (n = 16 fertile phases). Due to infrequent fecal sampling in February 2009 (i.e. the end of the mating season), the exact timing of fertile phases could not be determined for the remaining two females (fs13 and fs14; Table 1) which are therefore not included in some further analyses. However, both females conceived in the 2^nd^ week of February 2009, and furthermore, our hormone data (i.e. frequent sampling from October to January) revealed that both females conceived during their first cycle. Conceptions occurred 28 to 98 days after the start of the mating season.

### Female receptivity relative to reproductive state

**Figure 1:**
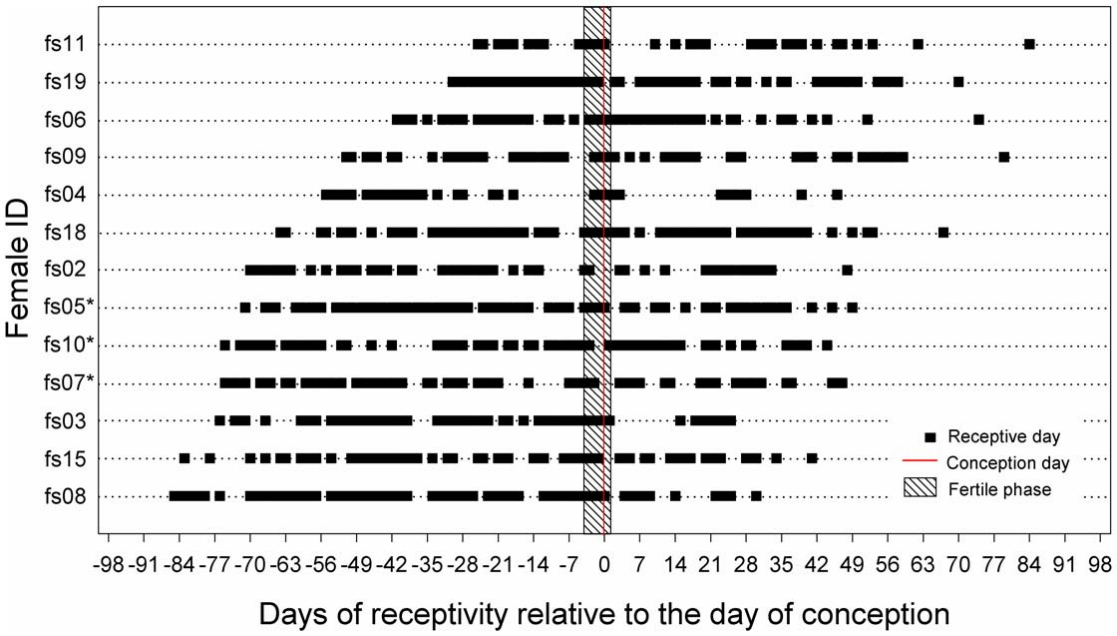
Female receptivity in relation to the hormonally determined timing of conception. Females marked with an asterisk conceived during the second, others during the first cycle of the mating season. Black squares indicate receptive days. The shaded area denotes the females' fertile phase.

**Figure 2:**
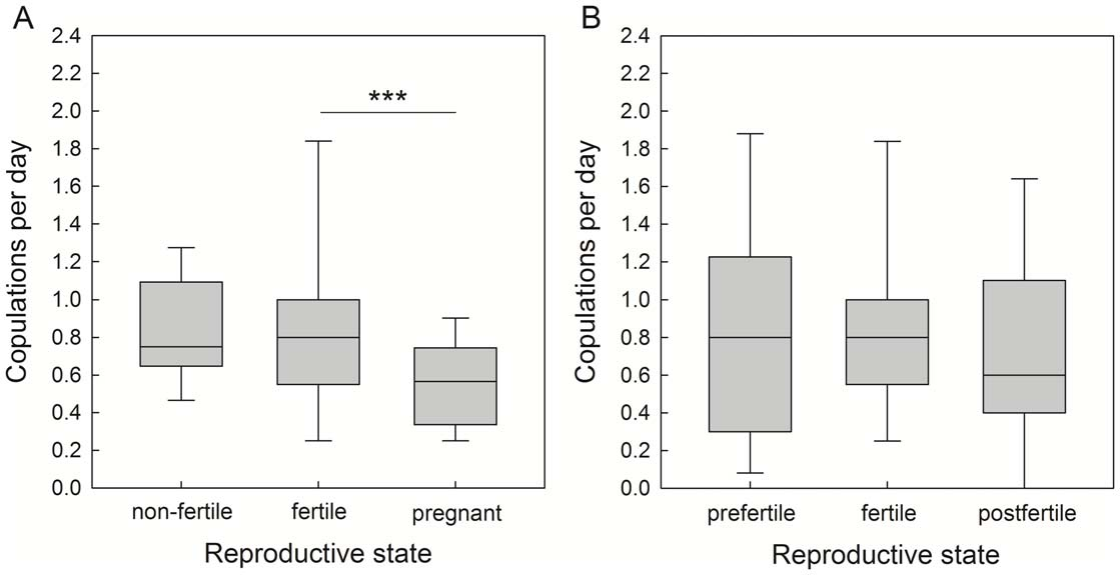
Female daily copulation frequencies in relation to reproductive state. (A) during the entire mating season, including non-fertile, fertile, and pregnant stages (n = 13 females, two mating seasons), and (B) within conception cycles, i.e. during the prefertile, fertile, and postfertile phase (i.e. the five days preceding the fertile phase, the five-days fertile phase, and the five days following the fertile phase). For detailed definitions see methods. The boxes indicate medians (line) and upper and lower quartiles. The whiskers indicate the 90th and 10th percentiles. ***: p<0.001.

Although females mostly conceived in their first ovarian cycle, they mated throughout the entire 4-month mating season (Figure 1). On 87% of days there was more than one female receptive (MS 07/08: 89%, range of females being receptive on the same days: 0–7; mean±sd: 3.0±1.7; median: 3; 7 females; MS 08/09: 85%, range: 0–7; mean±sd: 3.1±1.6; median: 3; 8 females). Mean duration of receptivity ( = number of days between the first and the last observed copulation within the mating season) was 115.4±7.3 days in 07/08 (n = 7) and 120.9±12.6 days in 08/09 (n = 8), respectively. Accordingly, females not only mated during their ovarian cycle(s) but also during acyclic and pregnant stages. In total 93.7% of copulations (n = 1053; 13 females) were recorded during non-fertile stages (58.3% before conception, and 35.4% during pregnancy), the remaining 6.3% occurred during female fertile phases. In a GLMM we found that female mating activity varied throughout the mating season, with daily copulation frequencies being significantly higher during fertile (*fert*, n = 62 observations; dummy coding reference category) stages than during pregnancy (*p*, n = 682 observations), but no significant difference was found between non-fertile (*n*, n = 749 observations) and fertile phases (*n* vs. *fert*: z = −0.70, p = 0.48; *p* vs. *fert*: z = −3.33, p<0.001; 13 females; Figure 2A). Female dominance rank was found to influence mating activity, with high-ranking females having higher daily copulation frequencies (z = −3.41, p<0.001).

To investigate within-cycle differences in daily copulation frequencies, i.e. between the prefertile (*pre*, n = 65 observations), fertile (*fert*, n = 62 observations; dummy coding reference category), and postfertile (*post*, n = 65 observations) phase of the conception cycle (as a potential indicator of behavioral estrus within cycles), we performed a second GLMM and found no significant differences between any of the three stages (*pre* vs. *fert*: z = −0.53, p = 0.60; *fert* vs. *post*: z = −0.91, p = 0.36; 13 females; Figure 2B).

### Matings

Out of 1180 copulations with information on ejaculation 89.4% were ejaculatory. Only 7.2% of copulations were preceded by the female presenting to the male (MS 07/08: 5.9%, n = 101; MS 08/09: 7.8%, n = 192), out of which only one occurred during a female's fertile phase. Copulations preceded by female presenting behavior occurred between days −113 and +54 relative to the timing of conception. Of all copulations for which approaches were known, 69.7% were initiated by the female (n = 278, median: 73.3%, range across females: 39.3%–100.0%; no female rank effect: Spearman's rho = −0.065, p = 0.82, n = 15). We found no significant differences in female initiation of copulations with regard to male rank (alpha and beta versus lower ranking; Wilcoxon signed ranks test: z = −0.369, p = 0.71, n = 15) and age (adult versus large subadult; Wilcoxon signed ranks test: z = −0.682, p = 0.50, n = 15). Mean number of matings per female during the entire mating season was 84.7±30.9 in 07/08 (range: 37–127, median: 73.0) and 85.9±31.8 in 08/09 (range: 39–144, median: 84.5). Daily copulation frequencies did not differ between multiparous (n = 11) and primiparous (n = 4) females (Mann-Whitney U-test: U = 12, p = 0.23).

### Mating partners

**Figure 3:**
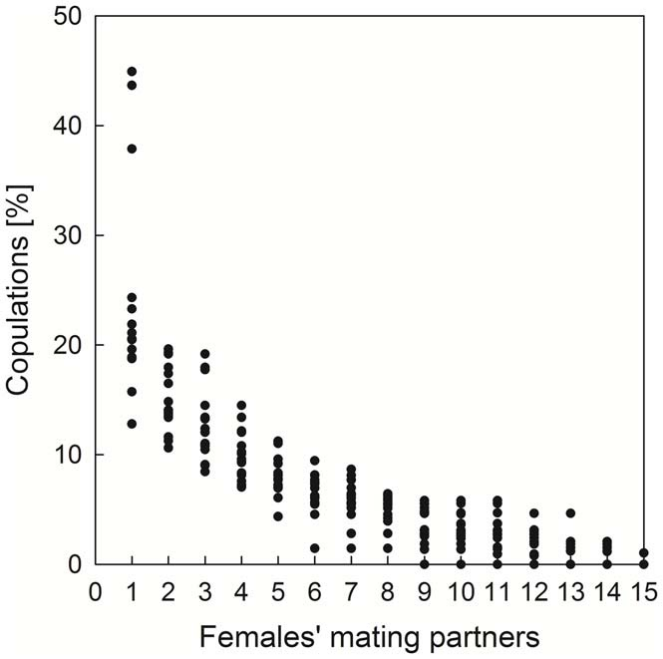
Distribution of copulations across the females' mating partners. Data are combined for two mating seasons (MS 07/08: 7 females, 13 males; MS 08/09: 8 females, 15 males). Note that not all females mated with all males, and that males are ranked separately for each female, i.e. numbers do not denote individuals, e.g. 1 = the most mated male for each female (including 8 different, high-and low-ranking, adult and large subadult males), 2 = the second most mated male, etc.

Out of 211 possible male-female mating dyads 185 have been observed. Thus, on average, females mated with 87.5±10.6% of all present adult and large subadult males (range: 61.5%–100%; median: 92.3; n = 15; number of mating partners per female: MS 07/08: mean±sd: 11.0±1.5; MS 08/09: mean±sd: 13.5±1.4). Female dominance rank was not significantly correlated with the number of mating partners (Spearman's rho = −0.115, p = 0.68, n = 15). However, female copulations were non-randomly distributed across male mating partners (Figure 3: ranked separately for each female; two mating seasons: MS 07/08: 7 females, 13 males; MS 08/09: 8 females, 15 males). With each female, many males (mean±sd: 8.8±2.4, range: 4–12, median: 9) obtained low (<10%) mating proportions, and only a few males (mean±sd: 3.4±1.0, range: 2–5, median: 3) obtained higher (>10%) proportions. The category “most mated male”, i.e. the male a given female copulated most often with, included 8 different, high- and low-ranking male individuals (MS 07/08: 5 different males, n = 7 females; MS 08/09: 6 different males, n = 8 females), indicating no general mating bias towards certain (e.g. top-ranking) males. The most mated male received 24.3%±9.8% of a female's total copulations (range: 12.8%–44.9%; median: 20.6%; 15 females). Skew calculations revealed that these inequalities did not occur by chance. The average B index across all females equaled 0.05±0.04 (n = 15; range: 0.001–0.139) and, overall, was significantly greater than expected by chance (p<0.001). Specifically, significant mating biases were found in 12 females (11 females: p<0.001; 1 female: p<0.02), two females showed a trend towards a mating bias (fs11: p = 0.06 and fs04: p = 0.08, respectively), and for only one female matings were distributed randomly across males (B index not significant; fs15: p>0.3).

Females mated with, on average, 2.2 males (range: 1–4) during the fertile phase of their conception cycle (n = 13 females). Only 3 females mated most frequently with their most mated male during the fertile phase of the conception cycle (all 3 were in consort with the alpha male; n = 11 females with significant mating skew). An analysis of within-cycle matings for non-consorted (i.e. not largely monopolized) females revealed that females did not mate more often with high-ranking males (i.e. the two top-ranking males) during the fertile phase compared to the pre- and postfertile phase (Friedman test: χ^2^ = 3.82, df = 2, n = 6, p = 0.15). Four of these females never mated with one of the two top-ranking males during their fertile phase.

### Consortships

**Figure 4:**
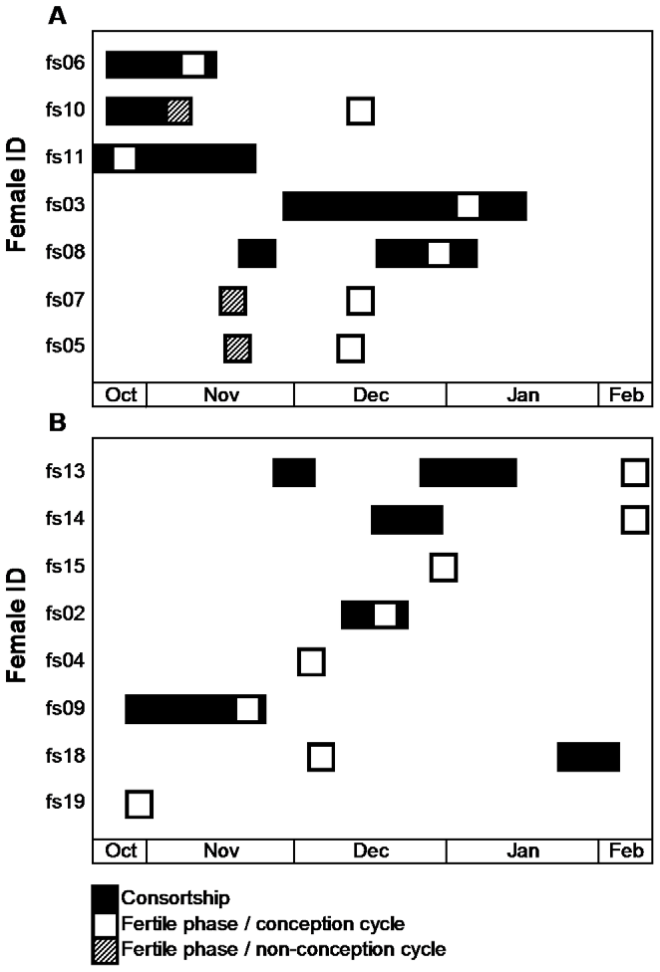
Sexual consortships in relation to the timing of hormonally determined female fertile phases. Data are presented for MS 07/08 (A) and MS 08/09 (B). White boxes indicate conception cycles and shaded boxes non-conception cycles, respectively.

Two thirds of the 15 females engaged in long sexual consortships (n = 12), ranging from 7 to 49 days, with one of the three top-ranking males (Figure 4). The consorting pair usually stayed within the main group, and some females occasionally sneaked copulations with other males. We found a non-significant trend for consorted females to have less mating partners (median: 1.0) during fertile phases of conception cycles compared to non-consorted females (median: 3.0; Mann-Whitney-U-test: U = 9.5, n_1_ = 6, n_2_ = 7, p<0.08). Of the 12 observed long consorts, five occurred during non-fertile stages (4 during acyclic stages: fs08, fs13, and fs14, Figure 4; 1 during pregnancy: fs18, Figure 4B). Only 6 consorts covered the female's fertile phase of the conception cycle, and one female was consorted during the fertile phase but did not conceive (Figure 4). Thus, taken together, in 9 cases (n = 15 females), females were not consorted by a male around the time of conception.

## Discussion

In this first study on female sexuality in wild Assamese macaques, we show evidence for: concealed fertility, extended female sexuality, and female promiscuity. However, copulations were non-randomly distributed across males irrespective of male rank. The potential implications of these rather unusual results shed new light on female reproductive strategies and deserve further discussion.

In contrast to most mammalian species [64], [65], Assamese macaque mating frequencies did not differ among prefertile, fertile and postfertile phases of the ovarian cycle suggesting that female reproductive hormones do not play a major role in influencing female attractivity and sexual activity around ovulation. This, together with the lack of apparent visual or auditory manifestations of female fertility, and the fact that the majority of fertile phases was not monopolized by dominant males (see below), suggests that fertility is concealed in this species (without implying an “active” concealment). We cannot completely exclude the possibility, however, that olfactory cues may provide certain information on female reproductive status (e.g. [42]), although the mating data suggest that, if such cues do exist, their precision in signaling the female fertile phase is probably poor. Ejaculate production and ejaculatory matings are energetically costly [66], [67], and frequent ejaculations can result in a substantial decrease in sperm number per ejaculate [68], [69], which may compromise fertility [68]. Thus, if males were able to assess female fertility precisely, one would expect them to focus their reproductive effort to the female fertile phase (e.g. [57]), and dominant males to monopolize fertile matings which, however, was not the case (also reflected in low alpha male paternity outcome; [49]). Instead, male Assamese macaques invest energy, time and sperm (89% ejaculatory copulations) in matings with females that cannot conceive, i.e. in females outside their fertile phase (94% non-procreative mating), suggesting that they are unable to discern when a female is fertile.

Usually, in most cercopithecine primates, females display a variety of cues that males may use to assess female ovarian state, with only a few potential exceptions, i.e. species in which ovulation is potentially concealed (e.g. *Semnopithecus entellus*: [22]; but see [70]; *Cercopithecus aethiops*: [71]; but see [72]). Among macaques, Tibetan macaques (*Macaca thibetana*), the Assamese macaque's closest relatives [73], show very similar mating and consort patterns (see below) but, unfortunately, no accompanying endocrine data are available so that the relationship between these behavioral patterns and the timing of ovulation remains unexplored [74].

Strong support that female fertility in Assamese macaques is undisclosed to males comes from the pattern of sexual consortships, i.e. close spatial associations between a male and a female that usually serve female mate-guarding by males around ovulation as seen in many primate species ([22], [38], [56], [70]; for review see [34]). Here, we recorded long sexual consortships (ranging from one up to six weeks) between only some of the females and high-ranking males [48], but in the majority of conception cycles (60%) females were not consorted at all by a male. Consorts occurred during all reproductive stages (including non-fertile ones), indicating that males probably cannot discriminate between female reproductive states differing in fitness relevance to males. If males were able to detect when females are likely to ovulate, we would, contrary to our results, expect consortships to be shorter, and to be linked to female fertile phases, and not to occur during acyclic stages (see [34], [75]). Further, although some female fertile phases overlap (which may diminish male monopolization potential), we would expect a higher proportion of consorted fertile phases, given their temporal distribution. Under such conditions of apparent inability of males to discern female fertility, high ranking males may choose to make the “best of a bad job” by consorting a few females for extended periods to secure at least some paternities [48].

Generally, females could benefit from concealed fertility whenever they receive male-delivered benefits through extended sexuality [76]. As previously shown, female Assamese macaques do not exhibit regular ovarian cycles outside the mating season, and mostly conceive during their first cycle [46]. Nevertheless, irrespective of the timing of conception, female mating activity was extended over the entire mating season, i.e. all females were receptive long before the onset of cyclic ovarian activity (up to three months before ovulation), and females continued mating upon conception, i.e. during the first 2–3 months of pregnancy, resulting in a considerable amount of non-procreative mating (c.f. [30]). This is in line with our conclusion that female mating activity in Assamese macaques is not under cyclic hormonal control.

In principle, extended female sexuality serves to obtain benefits from males (either from primary partners within pair-bonds or from multiple males [3], [18], [30], [76]–[78]). It has long been argued that in potentially infanticidal species females benefit from mating with multiple males by reducing the chance that males attack their future offspring [6], [7], [18], [20]. Infanticide is extremely costly for female primates, and in many species females exhibit prolonged mating periods (within ovarian cycles) and engage in situation-dependent receptivity [6], [18]. Concurrently, in numerous primate species females exhibit cues of ovulation probability to overcome the “female dilemma”, i.e. to bias paternity in the dominant male to protect their future offspring, and simultaneously dilute it among all males to prevent infanticidal attacks [25], [28], [35], [36].

Assamese macaque females lack overt cues to fertility, exhibit extended sexuality, and are highly promiscuous. But why not choose the dominant male? Investing heavily in diluting paternity among resident males instead of concentrating it in the dominant male (assuming him to be the best infant protector) may be favorable for females if (1) alpha male tenure is short (resulting in a shorter period to protect the infant), and (2) if the risk of infanticide mainly arises from within the group (by a resident male which rises to alpha position) instead of from an immigrant male taking over the alpha rank [33], [79]. Reproductive skew is significantly reduced in inside- compared to outside-takeover species, reflecting female tendencies toward polyandry in inside-takeover species [33]. Empirically investigating the effects of takeover modes requires comparative long-term data which are generally not available for many species including Assamese macaques. Given the low mating and paternity skew in our study population [48], [49], we may expect a high probability of takeover from inside and consequently also large benefits from paternity dilution in Assamese macaques.

Obviously, prolonged mating periods facilitate promiscuity but the extreme extended female sexuality observed in this study is puzzling, and it appears unlikely that females “need” four months to mate with each male in order to achieve maximum paternity dilution, hence suggesting other non-mutually exclusive functions than raising benefits from multiple males. In human females, for example, extended sexuality appears to enhance benefits from primary partners within pair-bonds [76], [77]. Is it possible that extended female sexuality in Assamese macaques has a similar function? Interestingly, although females copulated with virtually all males, matings were distributed non-randomly across males, i.e. females mated with many males on few occasions, while at the same time frequently copulating with only a few males irrespective of male rank. Almost every female had a different most frequent mating partner including high- and low-ranking, adult and large subadult males who obtained on average a quarter of a female's total copulations. Thus, we envision that extended sexuality in Assamese macaques may also function to obtain more direct benefits from primary partners. In other words, by frequently copulating with a particular male during a prolonged period of time (facilitated by concealed fertility), females and males may create special relationships despite and within the promiscuous mating system.

Male Assamese macaques appear to have no information on female fertility state (see above), and thus, in order to assess paternity certainty, cannot temporally relate matings to ovulation probability. Accordingly, mating frequencies may be their only estimate, hence function as probabilistic cues of perceived paternity (see [80]). Future studies are needed to test whether male-infant interactions (male Assamese macaques frequently interact with, protect and care for infants; Ostner & Schülke, unpub. data; Fürtbauer, pers. obs.) and/or male-female interactions outside the mating season reflect these non-dominance based mating biases (e.g. [80]–[82]). Also, it remains to be investigated whether mating biases indicate female preferences or are caused by male coercive behavior (see [83], [84]). Furthermore, genetic analyses are needed to determine whether primary partners are genetic fathers, and whether female mating biases are related to genetic traits (e.g. major histocompatibility complexes; e.g. [85], [86]).
